# Use of the Bonfils Intubation Fiberscope in Patients with Limited Mouth Opening

**DOI:** 10.1155/2012/297306

**Published:** 2012-08-21

**Authors:** Nabil A. Shollik, Sami M. Ibrahim, Ahmed Ismael, Vanni Agnoletti, Emanuele Piraccini, Ruggero Massimo Corso

**Affiliations:** ^1^Anesthesia Department, HMC-Weill Cornell Medical College, P.O. Box 24144, Doha, Qatar; ^2^Anaesthesia and Intensive Care Section, Department of Emergency, G.B. Morgagni-Pierantoni Hospital, viale Forlanini 34, 47100 Forlì, Italy

## Abstract

Airway management of patients with very limited mouth opening remains a challenge for the anaesthetist. We describe the use of the Bonfils Intubation Fiberscope for awake intubation in two patients with a very limited mouth opening. In the first case, a 60-year-old 80 kg female, scheduled for a right modified radical mastectomy for infiltrating ductal carcinoma (15 mm mouth opening, a short thick neck, limited neck extension, and a Mallampati class 4 airway), the Bonfils was advanced via the retromolar technique. In the second patient, a 34-year-old male, scheduled for a surgical tracheotomy for right tonsillar cancer, due to a neoplastic infiltration of the right temporomandibular joint (7 mm mouth opening and limited neck movement), the Bonfils was advanced using the midline approach. The Bonfils is a reusable, rigid, straight fiberoptic device with a curved tip, is 5 mm in diameter, and has several advantages: it is quick and easy to use, more cost effective than a flexible fiberscope, and is safe in expert hands, thanks to its smaller diameter. Our conclusion is that awake BIF intubation is a reliable, atraumatic, and well-tolerated procedure to secure a safe airway in patients with a limited mouth opening.

## 1. Introduction

Airway management of patients with very limited mouth opening remains a challenge for the anaesthetist. The standard approach for predicted difficult intubation is awake intubation with a flexible fiberscope [1]. This procedure however, may prove very difficult in patients with a distorted airway; it requires a lot of practice and is quite expensive [2].

## 2. Case Presentation

We describe the use of the Bonfils Intubation Fiberscope (BIF) (Karl Storz Endoscope, Tuttlingen, Germany) for awake intubation in two patients with a very limited mouth opening. The first was a 60-year-old 80 kg female (body mass index, BMI: 33 kg m^−2^) with an American Society of Anaesthesiology (ASA) physical status II, scheduled for a right modified radical mastectomy for infiltrating ductal carcinoma. Preoperative examination revealed a 15 mm mouth opening, a short thick neck, limited neck extension, and a Mallampati class 4 airway. In the operating room, she received midazolam 2 mg IV and a topical anaesthesia of the oropharynx with lidocaine 10%. The patient was administered oxygen (4 L min^−1^) for 5 minutes and then through the side port of BIF. The BIF (preloaded with a 7.0 mm cuffed tracheal tube, TT) was advanced via the retromolar technique. When it was positioned in front of the vocal cords, the TT was advanced over the scope. End-tidal capnography confirmed the tracheal placement and then a routine IV induction was performed. The second patient was a 34-year-old male (BMI: 16 kg m^−2^) with an ASA physical status I, scheduled for a surgical tracheotomy for right tonsillar cancer. The airway evaluation revealed a limited mouth opening (7 mm) due to a neoplastic infiltration of the right temporomandibular joint, as well as limited neck movement. Topical anaesthesia of the oropharynx using lidocaine 10% spray and local laryngeal anaesthesia by cricothyroid injection of 3 mL of lidocaine 2% were administered. He received IV fentanyl (100 *μ*g) and supplemental oxygen (4 L min^−1^) for 5 minutes, then through the side port of the BIF. A BIF preloaded with a 6 mm cuffed TT was advanced using a midline approach. When the BIF was in front of the vocal cords, the TT was advanced over the scope into the trachea. After confirmation of correct placement by capnography, a routine IV induction of anaesthesia was performed.

## 3. Discussion

**Figure 1:**
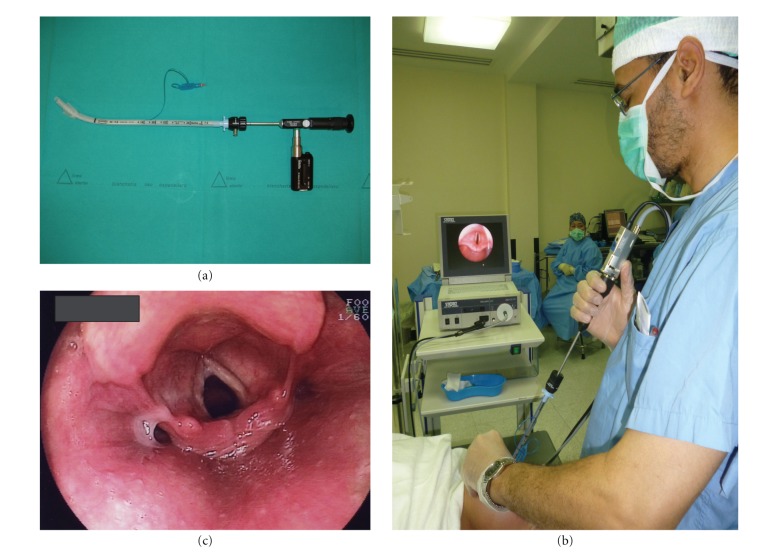
(a) Battery-powered Bonfils Intubation Fiberscope armed with an 7.5 mm inner diameter endotracheal tube, (b) intubation with a Bonfils intubation fiberscope. The video camera is attached at the eyepiece of the scope, (c) an endoscopic view of laryngeal aditus using Bonfils Intubation Fiberscope.

The BIF is a reusable, rigid, straight fiberoptic device with a curved tip and is 5 mm in diameter (Figure 1). It has been successfully used in patients with both predicted and unpredicted difficult airways [3, 4]. It has several advantages: it is quick and easy to use, more cost effective than a flexible fiberscope, and is safe in expert hands [5].

It has a distinct advantage over the latest videolaryngoscopes, which are larger in size and therefore, for patients with a poor mouth opening, it may be difficult to insert the blades or to pass the TT. The BIF, thanks to its smaller diameter, facilitates intubation in such patients. 

Our conclusion is that awake BIF intubation is a reliable, atraumatic, and well-tolerated procedure to secure a safe airway in patients with a limited mouth opening.
